# Chromosome-Scale Genome Assemblies of Two Korean Cucumber Inbred Lines

**DOI:** 10.3389/fgene.2021.733188

**Published:** 2021-11-19

**Authors:** Kihwan Song, Younhee Shin, Myunghee Jung, Sathiyamoorthy Subramaniyam, Keun Pyo Lee, Eun-A Oh, Jin Ho Jeong, Jeong-Gu Kim

**Affiliations:** ^1^ Department of Bioresources Engineering, Sejong University, Seoul, South Korea; ^2^ Research and Development Center, Insilicogen Inc., Gyeonggi-do, South Korea; ^3^ Genomics Division, National Institute of Agricultural Sciences, Nongsaengmyeong, Jeonju, South Korea

**Keywords:** Korean cucumber, genome, kimchi, slicer, Cucumis sativus

## Introduction

Practicing traditional food habits and using traditional ingredients are of major importance for maintaining a diet with good nutritional value. South Korea is well known for its fermented foods, particularly *banchan* (fermented side dishes such as kimchi), which are deeply rooted in Korean food culture. Moreover, Korean cuisine has unique characteristics that are widely accepted to provide various health benefits (Kim et al., 2016), with Korean food culture involving high consumption of vegetables due to the characteristics of its long agricultural history. According to the Korean Ministry of Agriculture definition of the standards and fundamentals of Korean food, one major constraint is that only food prepared with ingredients produced or cultivated in Korea can be considered Korean food. For example, kimchi prepared from imported Chinese cabbage cannot be considered Korean food; the same applies to other *banchan*. As part of the process of preserving Korean food culture, we have initiated the development of genetic resources for Korean varieties of cucumber (*Cucumis sativus* var. *sativus* L.), which is widely cultivated in Korea for both fresh and processed consumption. Cucumber originated in India and spread to other parts of the world through adaptation to various environmental factors and indigenous food habits (Sebastian et al., 2010). This process has led it to become the sixth most widely cultivated vegetable crop in the world, with 2.1 million hectares under cultivation (FAOSTAT, 2020). South Korea is the 16th largest producer of cucumber in the world, with three major cultivar groups being grown: the Baekdadagi-type, Nakhap-type, and Gasi-type cultivars (Park et al., 2021). In this study, we aimed to obtain detailed insights into the genetics of cucumber varieties by constructing chromosome-scale genome assemblies for two Korean cucumber inbred lines: JEF (semi-white Baekdadagi-type, mainly used for kimchi and other fermented foods) and KWS (Korean solid green, Nakhap-type, a slicer used fresh for salads and *gimbap* or Korean cold noodles).

As shown by previous studies of model plants and crops, a single reference genome is inadequate to capture the variation among different genetic lineages. For example, significant structural variation among maize inbred lines has been identified through analysis of multiple genomes (Tao et al., 2019). Furthermore, the cost of assembling multiple genomes has been significantly reduced by third-generation sequencing technologies and computational methods, leading to the construction of chromosome-scale genome assemblies for various crops with the aim of obtaining detailed insights into gene–trait associations (Yang et al., 2019). The first version of the cucumber draft genome was released in 2009 for inbred line 9,930, a lineage of the ‘Chinese Long’ cultivar (Huang et al., 2009); the genome has since been updated to version 3 (Li et al., 2019) and the chromosomal level Northern American cucumber genome published in 2012 (Yang et al., 2012). Further insight into variations among and within varieties has recently been provided by the publication of information on the genome of the pickling cucumber “Borszczagowski” (line B10) (Osipowski et al., 2020). As the chromosome-scale haploid genome assembly of “Chinese Long” line 9,930 (2n = 2x = 14, haploid number 7) is readily available to the public, we used it as our reference for the construction of chromosome-scale assemblies for the two Korean highly inbred lines.

## Value of the Data

These new genomes will serve as an additional genetic resource that can be used as a basis and reference for more detailed study into genetic variation and domestication history among Korean cucumber varieties. In addition, they may be valuable for conducting comparative analysis among and within the species in the genus *Cucumis*, which could improve the genome selection process in molecular-assisted breeding.

## Materials and Methods

### Sample Collection and Genomic DNA Extraction

**FIGURE 1:**
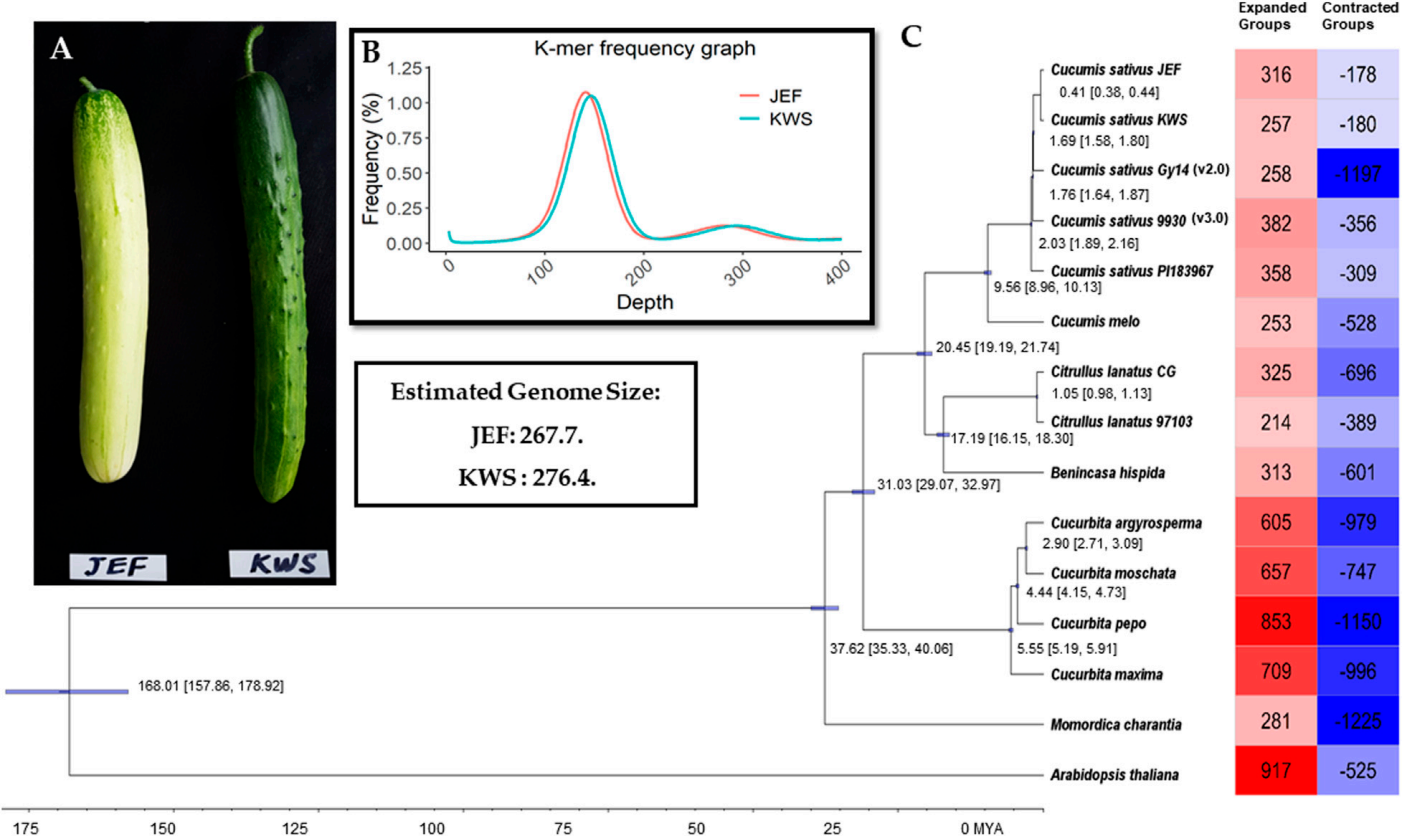
Summary of the sequencing. **(A)**. Cucumber fruits of the varieties sequenced in this article. **(B)**. Genome size estimation. **(C)**. Phylogenetic tree from the single-copy genes along with summaries of gene gain and loss.

The inbreed lines (i.e., JEF and KWS) are obtained from the leading varieties “Joeun Baekdadagi” and “Gyeoulsal-i Cheongjang” from Fomer Heungnong Seeds Co. After selecting the individual that best characteristics represent of each group in the F_2_ populations, two inbreeds were raised through self-fertilization. The resulted breed line i.e., JEF is gynoecious, which is semi-white fruit skin color with white spine and KWS is monoecious which is uniform dark green skin color with black spine (Figure 1A). The *Cucumis sativus* breeding line plants were directly harvested in June 2018 in a field in Wanju, Jeollabuk-do, South Korea (35°90′ N, 127°15′ E), near the National Institute of Agricultural Sciences. Sampled fruits are shown in Figure 1A, and the complete work flow followed in this study is given in Supplementary Figure S1.

### DNA Sequencing and *de novo* Genome Assembly

Total DNA was isolated from the samples individually according to sequencing protocols. The isolated DNA was sequenced using two different sequencing systems, PacBio Sequel (Pacific Biosciences, Menlo Park, CA, United States) and Illumina HiSeq 2,500 (Illumina, San Diego, CA, United States), which are widely used in long- and short-read sequencing. For Illumina sequencing, DNA was prepared using the TruSeq Nano DNA Library Prep Kit (Illumina). For PacBio sequencing, DNA was prepared using the SMRTbell Express Template Prep Kit (Pacific Biosciences; catalog no. 101–357–000). The experimental procedures were fully conducted by DNA Link (Seoul, Korea), an authorized service provider in South Korea. The Illumina paired-end sequences were initially subjected to filtering of technical artifacts (i.e., base-calling errors [Phred quality score ≤ Q20]) and adapters using Trimmomatic v. 0.32 (Bolger et al., 2014). These Illumina reads were used for error correction of PacBio reads in CLC Assembly Cell v. 5.1.1 (Qiagen, Hilden, Germany). The corrected PacBio reads were used to prepare the initial draft version of the cucumber genomes in FALCON-Unzip v. 0.30, a haplotype assembler program (Chin et al., 2016). Finally, using the RaGOO method (Alonge et al., 2019), the genome contigs were clustered and reordered according to their alignment with chromosomal units in the reference genome (‘Chinese Long’ 9,930). The assembled genomes were assessed for completeness using BUSCO v. 4.1.4 with the Viridiplantae_odb10 reference dataset (Seppey et al., 2019).

### Reference Mapping of Bacterial and Organelle Genes

To prepare a clean reference genome, it was necessary to remove bacterial contamination and organelle genomes from the database. The complete GenBank database, which contains draft and reference genomes of bacteria and organelles (mitochondria and plastids), was used as the reference to determine which reads should be removed from the raw sequences. All reference mapping of preprocessed reads was conducted using Bowtie 2 v. 2.2.8 (Langmead and Salzberg, 2012). Details regarding reference paths and sizes are given in Supplementary Table S1, and mapping statistics are given in Supplementary Table S2.

### Genome Size Estimation

All the Illumina-preprocessed sequences from the paired-end library were subjected to genome size estimation based on *k*-mers. The *k*-mer frequencies (*k*-mer size = 17) were obtained using Jellyfish v. 2.0 (Marçais and Kingsford, 2011), and the genome size was calculated from the following formulas: genome coverage depth = (*k*-mer coverage depth × average read length)/(average read length–*k*-mer size +1); genome size = total base number/genome coverage depth. Here, the *k*-mer coverage depth is the major peak of the *k*-mer distribution.

### Prediction and Classification of Repeat Regions

Repeat regions in the cucumber genomes were predicted using RepeatModeler (www.repeatmasker.org/RepeatModeler/) and classified into subclasses using the repbase v. 20.08 reference database (www.girinst.org/repbase/) (Bao et al., 2015). Finally, the repeats were masked in the genome using RepeatMasker v. 4.0.5 (www.repeatmasker.org) with RMBlastn v. 2.2.27+. The results are shown in Supplementary Figure S3.

### RNA Sequencing

The mRNA library from the collected samples was prepared according to the TruSeq Stranded mRNA Prep Kit protocol (Illumina). The isolated mRNA was sequenced using the Illumina sequencer (Supplementary Tables S4 and Supplementary Tables S5).

### Gene Prediction and Annotation

The genes from the cucumber draft genomes were predicted using an in-house gene prediction tool that includes three modules: an evidence-based gene modeler (EVM), an *ab-initio* gene modeler, and a consensus gene modeler. The Illumina-sequenced transcriptomes were mapped to the respective repeat-masked draft genomes using TopHat, and Trinity v2.5.1 method was used to assemble the transcripts and mark gene structural boundaries (Trapnell et al., 2012). The *ab-initio* gene modeler and EVM, which included Exonerate (Slater and Birney, 2005), Geneid and AUGUSTUS (Stanke et al., 2006), were trained with several genomes. The final gene and transcript models were optimized using a consensus gene modeler and annotated using Trinotate v. 3.0.1 (Bryant et al., 2017).

### Comparative Genome Analysis

Total proteins from the two cucumber genomes were subjected to ortholog analysis to provide insight into the differences between cucumber proteins and those of other plants. In total, 14 genomes from Cucurbitaceae (including the two assembled in this study) were used in the ortholog analysis, with Brassicaceae as outliers (Figure 1D and Supplementary Table S3). The complete proteins of the selected genomes were also subjected to ortholog analysis using OrthoMCL (Li et al., 2003). The single-copy genes from the given genomes were subjected to phylogenetic tree reconstruction using BEAST (Bayesian Evolutionary Analysis Sampling Trees) to assess the evolutionary time and the degree of similarity among the given genomes (Suchard et al., 2018). Furthermore, to assess the gain and loss of genes in the given genomes, the proteins were analyzed using CAFE v. 3.1 (Han et al., 2013).

### Preliminary Analysis Report

Initially, the sizes of the cucumber genomes were estimated to be 267.7 (JEF) and 276.4 MB (KWS) (Figure 1B) based on ∼50 GB of short-read sequences (Table 1A and Supplementary Table S4), but 230.8 MB (JEF) and 231.1 MB (KWS) based on the representative scaffolds assembled from ∼30 GB of error-corrected long-read sequences (Table 1A,B). The N50s of the assembled genomes were 30.5 MB (JEF) and 31.3 MB (KWS), and 40% of the assembled contigs were covered by repeats, in which the long terminal repeat (LTR) elements dominated, accounting for 36% of contigs (Supplementary Figure S3). In total, 25,968 genes were predicted from the JEF genome and 26,011 from KWS, with average sizes of 4,111 and 4,114 bases respectively, and BUSCO scores of 97.88 and 98.35% completeness respectively. (Table 1C). Finally, 66.54% of JEF genes and 65.96% of KWS genes had homologous sequences in GenBank, while 60.25% of JEF genes and 59.82% of KWS genes had gene ontology descriptions (Table 1D). The two genomes were scaffolded onto the reference “Chinese Long” 9,930 genome using the RaGOO method. Overall, these genome assemblies have ∼5 MB of additional bases compared with the reference and similar BUSCO completeness scores, indicating that they are of good quality. Additionally, an average of 99% of both DNA and RNA sequences were mapped to the reference assembly as an additional measure to ensure the quality of the new assemblies (Supplementary Figure S2). The ortholog analysis revealed genome-specific genes, as well as gain and loss of genes, in the selected cucumber genomes (Figure 1C and Supplementary Figure S4). In addition, the RNA samples were collected from five different developmental stages, revealing that both genomes contain genes expressed differentially in different organs or at different stages (Supplementary Figures S5 and Supplementary Figures S6).

**TABLE 1:** Summary of the sequencing to annotation of the cucumber draft genomes along with the reference.

	JEF	KWS	Cucumber_9,930_v3 (GCF_000,004,075.3)
(A) Sequencing			
Short Read	72.7 GB (315.10X)	75.9 GB (328.69X)	
Long Reads	31.7 GB (137.48X)	37.0 GB (160.38X)	
(B) Assembly			
Genome size estimation	267,736,921	276,372,239	—
Total length, bp	230,754,408	231,006,969	226,211,662
Total length/Estimation	86.19%	83.59%	—
No. of contigs	7 Chr +54 unplaced	7 Chr +57 unplaced	7 Chr +77 unplaced
Scaffold N50 (Contig N50)	30,569,742 (7,362,017)	31,270,087 (8,654,608)	31,125,843
N (%)	0.01%	0.01%	0.02%
GC (%)	33.23%	33.25%	32.82%
Repeats (MB)	93.47 (40.50%)	94.41 (40.87%)	
Repeats against references (9,930) 91.00 (39.44%)	91.00 (39.44%)	91.69 (39.69%)	84.02 (37.14%)–in our method
BUSCO	99.06%	98.82%	98.82%
(C) Structural annotations			
No. of genes	25,968	26,011	24,317
Average gene length (bp)	4,111.58	4,114.07	4,068.49
BUSCO (Viridiplantae)	97.88%	98.35%	100.00%
(D) Functional annotations			
No. hits	8,690 (33.46%)	8,853 (34.04%)	
Blast hits	17,278 (66.54%)	17,158 (65.96%)	
Gene Ontology	15,645 (60.25%)	15,561 (59.82%)	
KEGG	3,725 (66.54%)	3,718 (14.29%)	

## Data Availability

The datasets presented in this study can be found in online repositories. The names of the repository/repositories and accession number(s) can be found in the article/Supplementary Material.
