# Supplementary material for: Chromosome-Scale Genome Assemblies of Two Korean Cucumber Inbred Lines
Source: Front Genet. 2021 Nov 19;12:733188. doi: 10.3389/fgene.2021.733188 (PMC8640492; doi:10.3389/fgene.2021.733188)
Supplement: Supplementary file 1 [file Presentation1.PPTX]

## Slide 1
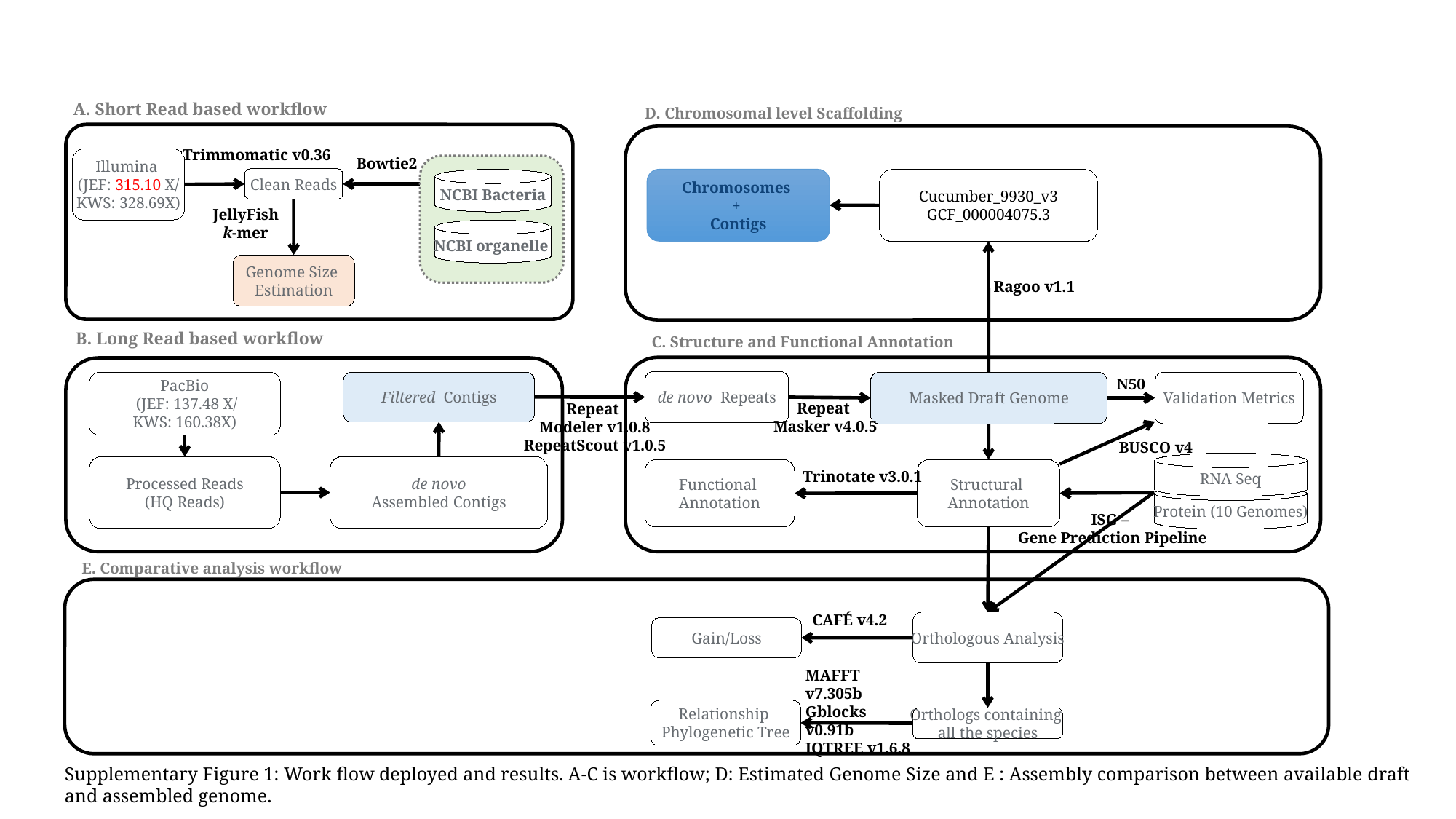

A. Short Read based workflow
D. Chromosomal level Scaffolding
Trimmomatic v0.36
Illumina
(JEF: 315.10 X/
KWS: 328.69X)
Bowtie2
NCBI Bacteria
NCBI organelle
Clean Reads
Chromosomes
+
Contigs
Cucumber_9930_v3
GCF_000004075.3
JellyFish
k-mer
Genome Size
Estimation
Ragoo v1.1
B. Long Read based workflow
C. Structure and Functional Annotation
de novo Repeats
N50
Masked Draft Genome
Validation Metrics
Repeat
Masker v4.0.5
BUSCO v4
Functional
Annotation
Structural
Annotation
Trinotate v3.0.1
Protein (10 Genomes)
ISG –
 Gene Prediction Pipeline
PacBio
 (JEF: 137.48 X/
KWS: 160.38X)
Filtered Contigs
Processed Reads
(HQ Reads)
de novo
Assembled Contigs
Repeat
Modeler v1.0.8
RepeatScout v1.0.5
RNA Seq
E. Comparative analysis workflow
CAFÉ v4.2
Orthologous Analysis
Gain/Loss
MAFFT v7.305b
Gblocks v0.91b
IQTREE v1.6.8
Relationship
Phylogenetic Tree
Orthologs containing
all the species
Supplementary Figure 1: Work flow deployed and results. A-C is workflow; D: Estimated Genome Size and E : Assembly comparison between available draft and assembled genome.

## Slide 2
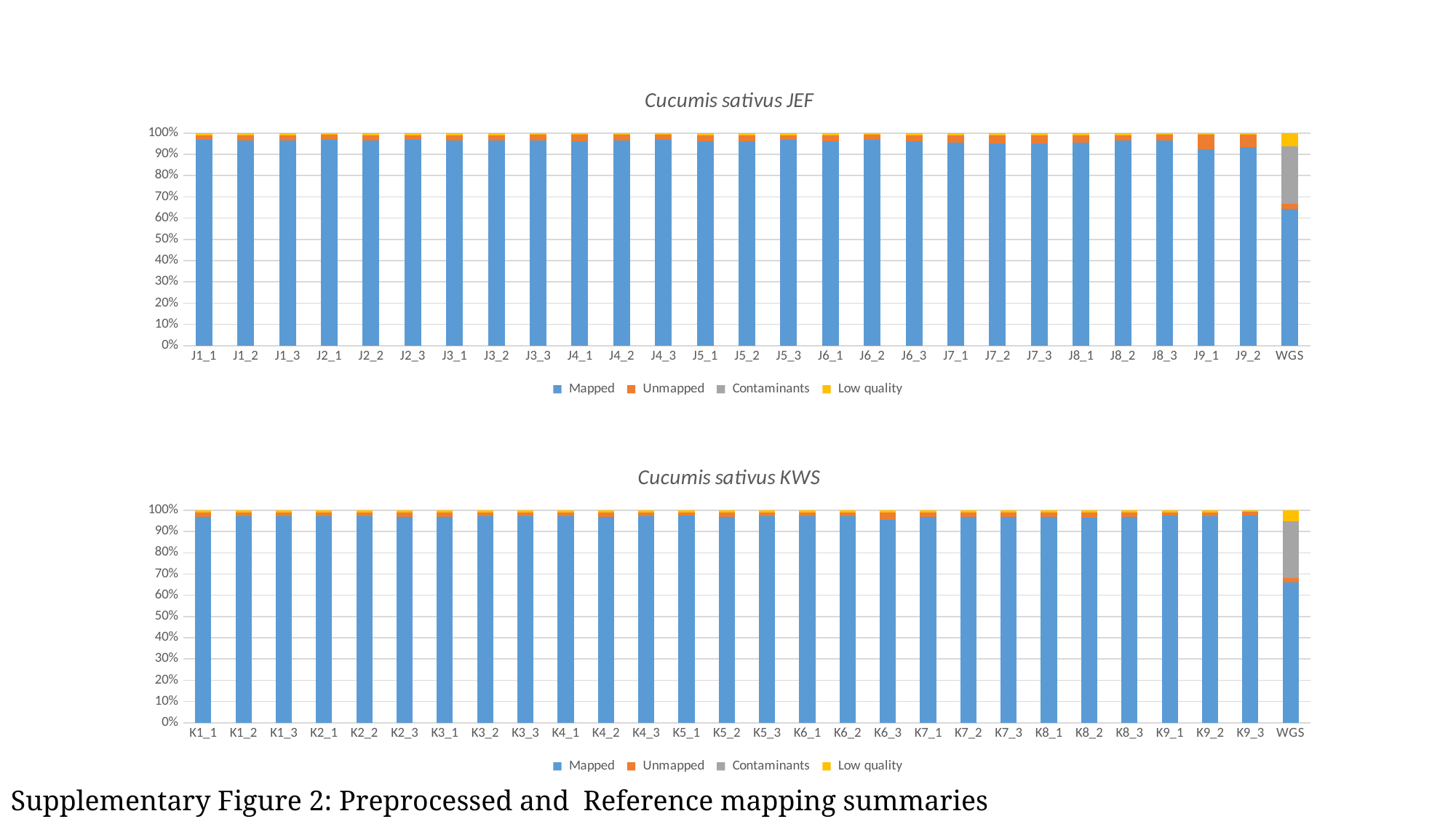

### Chart: Cucumis sativus JEF
| Category | Mapped | Unmapped | Contaminants | Low quality |
|---|---|---|---|---|
| J1_1 | 61876192.0 | 1407292.0 | None | 642260.0 |
| J1_2 | 51568030.0 | 1259639.0 | None | 530579.0 |
| J1_3 | 63246960.0 | 1509897.0 | None | 698789.0 |
| J2_1 | 52076370.0 | 1111398.0 | None | 507848.0 |
| J2_2 | 62442896.0 | 1660156.0 | None | 628980.0 |
| J2_3 | 77400480.0 | 1776728.0 | None | 807970.0 |
| J3_1 | 52812518.0 | 1373535.0 | None | 550275.0 |
| J3_2 | 68701732.0 | 1640401.0 | None | 733943.0 |
| J3_3 | 57855306.0 | 1564104.0 | None | 566964.0 |
| J4_1 | 63994440.0 | 1828427.0 | None | 631049.0 |
| J4_2 | 58306830.0 | 1474026.0 | None | 557020.0 |
| J4_3 | 76720528.0 | 1720384.0 | None | 699708.0 |
| J5_1 | 63810390.0 | 1794849.0 | None | 689311.0 |
| J5_2 | 54769108.0 | 1524190.0 | None | 611430.0 |
| J5_3 | 69681582.0 | 1667887.0 | None | 690415.0 |
| J6_1 | 54283086.0 | 1582671.0 | None | 621789.0 |
| J6_2 | 58653398.0 | 1403719.0 | None | 546275.0 |
| J6_3 | 66425722.0 | 1953393.0 | None | 730939.0 |
| J7_1 | 50551182.0 | 1771328.0 | None | 556596.0 |
| J7_2 | 54806188.0 | 2297540.0 | None | 573296.0 |
| J7_3 | 59990846.0 | 2343530.0 | None | 706932.0 |
| J8_1 | 47965394.0 | 1757875.0 | None | 514093.0 |
| J8_2 | 55297100.0 | 1396908.0 | None | 573034.0 |
| J8_3 | 76330692.0 | 2004856.0 | None | 741942.0 |
| J9_1 | 59264178.0 | 4231124.0 | None | 573596.0 |
| J9_2 | 48328022.0 | 2914921.0 | None | 477421.0 |
| WGS | 463362478.0 | 16857327.0 | 195347825.0 | 44350352.0 |
### Chart: Cucumis sativus KWS
| Category | Mapped | Unmapped | Contaminants | Low quality |
|---|---|---|---|---|
| K1_1 | 55005990.0 | 1210063.0 | None | 623227.0 |
| K1_2 | 62816884.0 | 1175424.0 | None | 626620.0 |
| K1_3 | 63249638.0 | 1095028.0 | None | 619886.0 |
| K2_1 | 64080114.0 | 1136322.0 | None | 625214.0 |
| K2_2 | 55774560.0 | 1065954.0 | None | 592966.0 |
| K2_3 | 55185470.0 | 1133441.0 | None | 588723.0 |
| K3_1 | 61040852.0 | 1376054.0 | None | 678498.0 |
| K3_2 | 62624698.0 | 1227564.0 | None | 648762.0 |
| K3_3 | 63951816.0 | 1204590.0 | None | 636812.0 |
| K4_1 | 52579014.0 | 1009156.0 | None | 550070.0 |
| K4_2 | 54864604.0 | 1167051.0 | None | 566309.0 |
| K4_3 | 55641072.0 | 1061738.0 | None | 567670.0 |
| K5_1 | 59083896.0 | 987093.0 | None | 559167.0 |
| K5_2 | 58979194.0 | 1193863.0 | None | 671301.0 |
| K5_3 | 60525298.0 | 1109690.0 | None | 623278.0 |
| K6_1 | 75010228.0 | 1365187.0 | None | 754577.0 |
| K6_2 | 58611554.0 | 1011875.0 | None | 581825.0 |
| K6_3 | 60098154.0 | 2156691.0 | None | 577577.0 |
| K7_1 | 64291856.0 | 1360185.0 | None | 625349.0 |
| K7_2 | 63658652.0 | 1410901.0 | None | 700559.0 |
| K7_3 | 63537852.0 | 1433935.0 | None | 703191.0 |
| K8_1 | 57767962.0 | 1309196.0 | None | 552868.0 |
| K8_2 | 56935634.0 | 1447779.0 | None | 587041.0 |
| K8_3 | 57772482.0 | 1332595.0 | None | 569289.0 |
| K9_1 | 55746202.0 | 894970.0 | None | 544092.0 |
| K9_2 | 63567700.0 | 1131447.0 | None | 648949.0 |
| K9_3 | 61512228.0 | 936827.0 | None | 553175.0 |
| WGS | 495341411.0 | 17757875.0 | 198790189.0 | 39889359.0 |Supplementary Figure 2: Preprocessed and Reference mapping summaries

## Slide 3
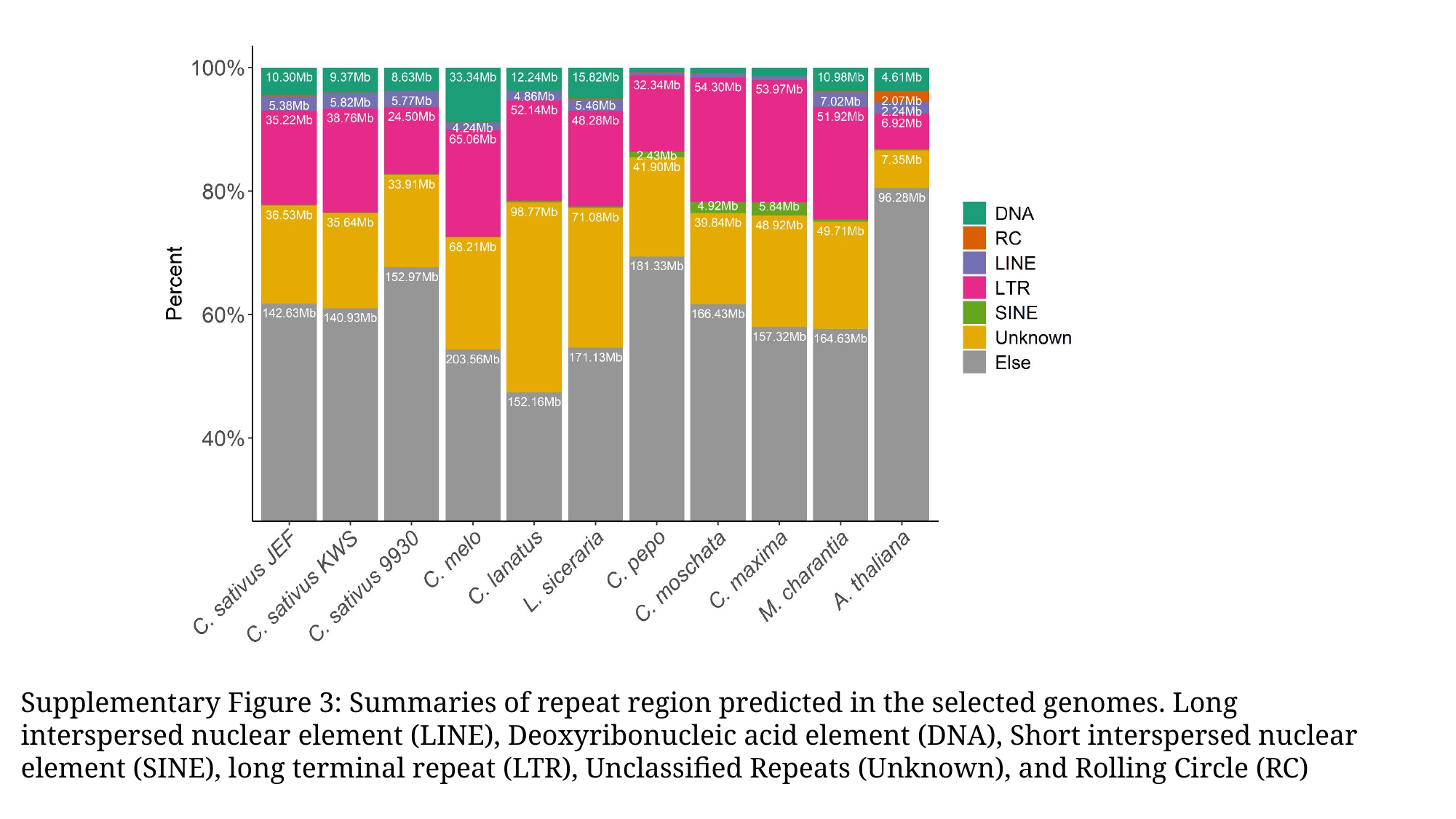

Supplementary Figure 3: Summaries of repeat region predicted in the selected genomes. Long interspersed nuclear element (LINE), Deoxyribonucleic acid element (DNA), Short interspersed nuclear element (SINE), long terminal repeat (LTR), Unclassified Repeats (Unknown), and Rolling Circle (RC)

## Slide 4
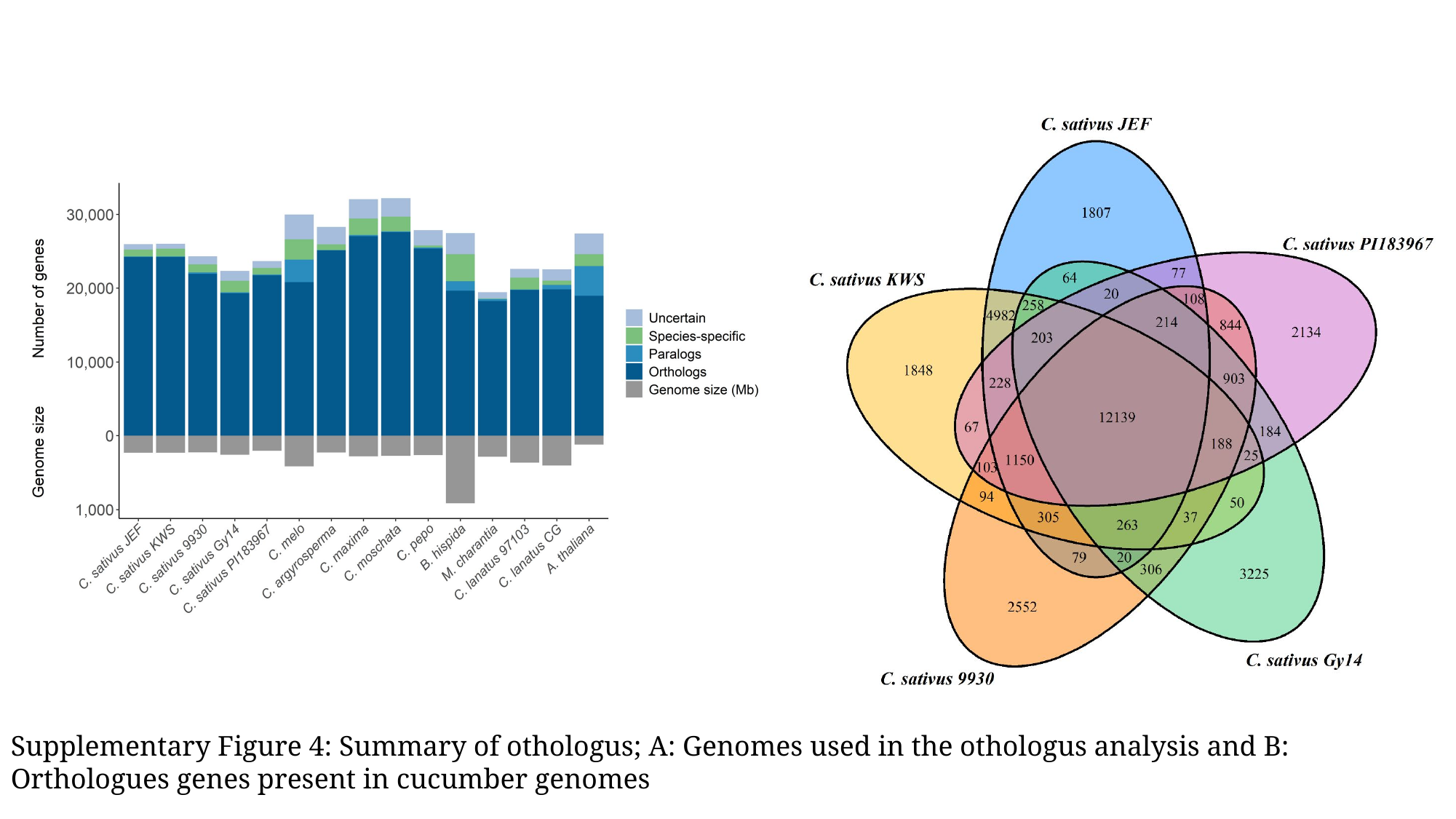

Supplementary Figure 4: Summary of othologus; A: Genomes used in the othologus analysis and B: Orthologues genes present in cucumber genomes

## Slide 5
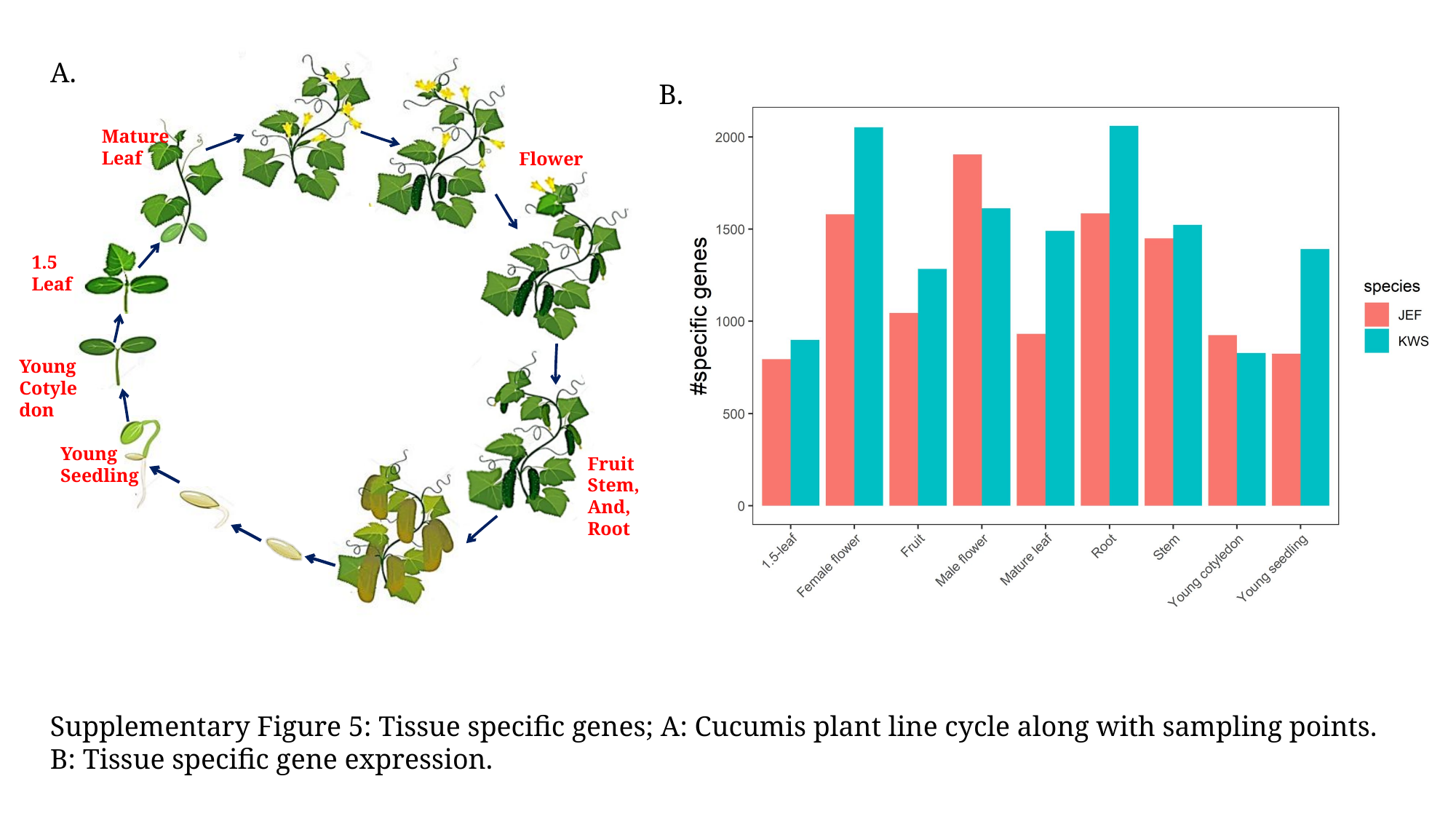

Mature
Leaf
Flower
1.5 Leaf
Young
Cotyledon
Young
Seedling
Fruit
Stem,
And,
Root
A.
B.
Supplementary Figure 5: Tissue specific genes; A: Cucumis plant line cycle along with sampling points. B: Tissue specific gene expression.

## Slide 6
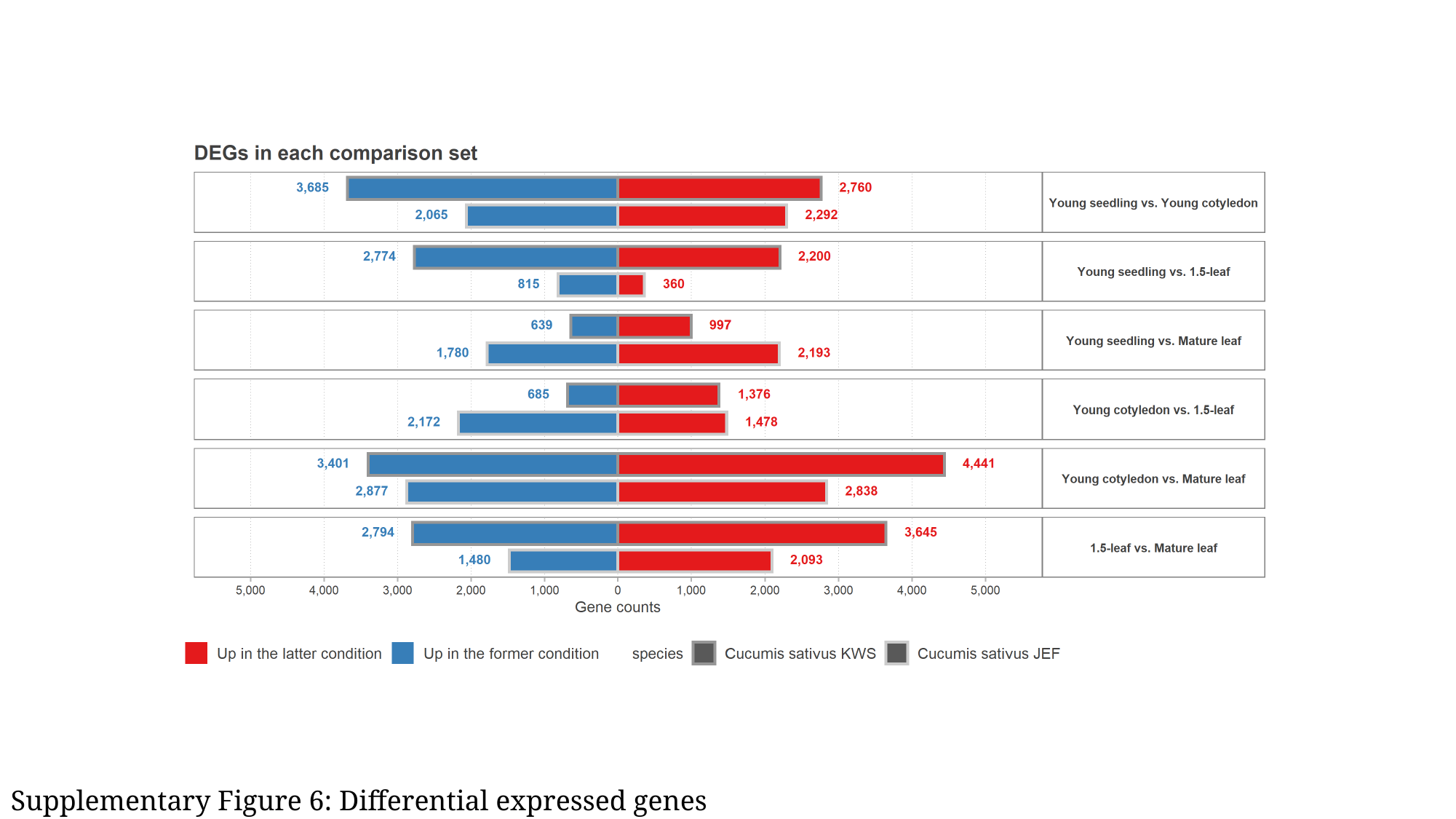

Supplementary Figure 6: Differential expressed genes
